# Genetically engineered mouse models of craniopharyngioma: an opportunity for therapy development and understanding of tumor biology

**DOI:** 10.1111/bpa.12501

**Published:** 2017-04-17

**Authors:** John Richard Apps, Juan Pedro Martinez‐Barbera

**Affiliations:** ^1^ Developmental Biology and Cancer UCL Great Ormond Street Institute of Child Health, University College London Guilford Street London WC1N 1EH UK

**Keywords:** craniopharyngioma, mouse model

## Abstract

Adamantinomatous craniopharyngioma (ACP) is the commonest tumor of the sellar region in childhood. Two genetically engineered mouse models have been developed and are giving valuable insights into ACP biology. These models have identified novel pathways activated in tumors, revealed an important function of paracrine signalling and extended conventional theories about the role of organ‐specific stem cells in tumorigenesis. In this review, we summarize these mouse models, what has been learnt, their limitations and open questions for future research. We then discussed how these mouse models may be used to test novel therapeutics against potentially targetable pathways recently identified in human ACP.

## Introduction

Craniopharyngiomas (CPs) are benign epithelial tumors of the sellar region that are associated with considerable morbidity and premature mortality 30. This is predominantly due to their tendency to cause damage to surrounding structures, leading to hypothalamic injury and associated obesity, visual deficits and pituitary dysfunction, including diabetes insipidus 35. This damage is often further augmented by the side effects of surgery and radiotherapy, the mainstay of clinical management 35.

Two subtypes have been defined; adamantinomatous craniopharyngioma (ACP), the commonest tumor of the sellar region in childhood, which frequently harbours mutations in the *CTNNB1* gene (encoding beta‐catenin), and papillary (PCP), predominantly a disease of adults, which frequently carry activating *BRAF p.V600E* mutations 30.

Considerable efforts have been invested in understanding the biology of craniopharyngioma to speed up the development of novel therapeutic strategies. For PCP, BRAF inhibitors have been found to have some benefit in the limited cohorts of patients published to date 6, 9. In contrast, no such novel, rationally targeted therapies have yet been successful for ACP.

This review summarizes the development and use of genetically engineered mouse models (GEMMs) in understanding the biology of craniopharyngioma and their potential use in developing future novel therapies. Specialized reviews covering clinical aspects, molecular pathology and the use of cell cultures and xenografts models are found in this special edition and elsewhere 5, 36.

## Why Use Genetically Engineered Mouse Models?

Mice have been used in studying the molecular biology of tumors for decades. Their relatively small size, quick reproduction times, and low maintenance costs make them particularly amenable for rapid testing of biological and therapeutic hypotheses in a manner and timescale not usually possible in human patients 14, 29. This is particularly the case for CP where, the relative rarity and chronic nature of the disease makes it difficult to study and has meant that there are currently no published randomized control trials of treatment to date.

A variety of methods have been used to model tumors in mice. For craniopharyngioma two approaches have been successful, xenografting and genetically engineered mouse models (GEMMs) 2, 10, 15, 42, 44. Xenografting of patient material either orthotopically or heterotopically into mice has enabled the direct study of human tissue in an *in vivo* setting and its use in craniopharnygioma is reviewed in a separate paper in this series (Stache and Holsken) 10, 42, 44.

In contrast, GEMMs utilize a range of techniques to manipulate the expression of genes and generate tumors of murine origin 18. Since their first description in the early 1980s, they have given insight in the mechanisms of tumor initiation, progression, interactions with the host and provided a key tool used in the development and testing of modern targeted therapeutics in a range of tumor types 14, 18. The ability to control the expression of individual genes at specific times and in specific cells/tissues enables detailed analyses of their *in vivo* function. This coupled with lineage tracing techniques, allowing the tracking of cell populations across time, has provided unique insights into the spatial organisation and regulation of tissues and tumors. Such approaches have improved our understanding of ACP biology, with perhaps wider implications for other tumor types 1, 2, 15. These will be discussed below.

The use of GEMMs in preclinical trials is enabling detailed study of the efficacy, pharmacology and pharmacogenomics of targeted therapies in an in vivo system, complementing in vitro data using cell lines. Whilst there have been notable successes using this approach leading to novel treatments for cancer patients, (e.g. the use of the smoothened inhibitor GDC‐0449 in a preclinical model of medulloblastoma, later validated in the clinic), there have been many disappointments, where promising results in the preclinical setting have failed to translate to benefit in patients with the disease 7, 14, 38. Reasons for these failures are multiple and may be case‐dependent. Sometimes, there are underlying biological differences, after all no mouse model can recapitulate the complexity of a human tumor wholly. For instance, the genetic heterogeneity and clonal evolution seen both within individual human tumors and between tumors of the same type can be difficult to model in mouse 18. However, it is generally recognized that the design of the preclinical research can have a critical impact and efforts must be done to model the human disease as closely as possible in the preclinical trials 14, 43. For instance, testing of new agents in mice often does not recapitulate how they will be applied to the patients, e.g. the survival benefit of a drug administered to naïve murine tumor may not translate to benefit to an extensively pretreated human tumor.

In our view, benign tumors such as craniopharyngioma, characterized by a low mutational load, may be easier to model in GEMMs and provided that preclinical trials are robust and well‐designed, data are more likely to be extrapolated to human patients. Nonetheless, GEMMs should be combined with other preclinical tools, including well‐characterized and validated cell lines and xenograft models. The critical point is to be aware of the limitations of the research model used and ensure that any aspect of the research being investigated, whether a novel pathway or a targeted treatment, is conserved between the GEMM and the human tumors.

## Similar Molecular Aetiology In Gemm Models and Human ACP

**Figure 1:**
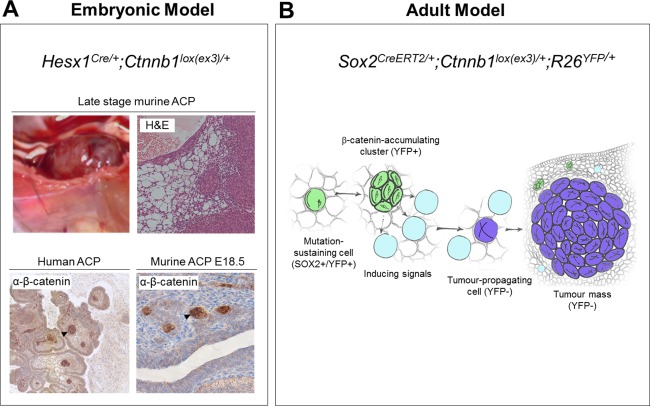
Genetically engineered mouse models of ACP. A) Embryonic model. Over‐activation of the WNT pathway in the developing pituitary results in large cystic/solid tumors. Clusters of cells accumulating nucleo‐cytoplasmic beta‐catenin (arrows) are present in both human ACP and murine (E18.5) pretumoral pituitaries. B) Inducible model. Tamoxifen‐induced activation of the WNT pathway at 6 weeks of age results in the formation of beta‐catenin accumulating clusters followed by tumor formation. However, lineage tracing with yellow fluorescent protein (YFP) shows that tumors are not derived from clusters and do not contain activated beta‐catenin suggesting a non‐cell autonomous mechanism of tumorigenesis. Clusters secrete numerous factors, e.g. SHH, BMPs, FGFs and inflammatory modulators potentially inducing tumorigenesis in a paracrine manner (Reprinted from *Cell Stem Cell*, **13**, Andoniadou CL, Matsushima D, Mousavy Gharavy SN, Signore M, Mackintosh AI, Schaeffer M, Gaston‐Massuet C, Mollard P, Jacques TS, Le Tissier P, *et al*., Sox2(C) stem/progenitor cells in the adult mouse pituitary support organ homeostasis and have tumor‐inducing potential, pp 433–445, Copyright 2013, with permission from Elsevier).

Two GEMMS of ACP have been developed, which we will call the embryonic model and the inducible model (summarized in Figure 1) 2, 15. No models of PCP have so far been developed. In both ACP models, cells express an oncogenic form of beta‐catenin (encoded by the *Ctnnb1* gene), which is functionally comparable to that identified in human ACP tumors. In the mouse models, expression of oncogenic beta‐catenin is achieved through cre‐recombinase‐mediated excision of exon 3, whilst human tumors harbor over‐activating mutations, mostly in exon 3 2, 12, 15, 22, 27, 39. The final outcome is the same in mouse and human ACP, the expression of a degradation‐resistant form of mutant beta‐catenin leading to the over‐activation of the WNT/beta‐ catenin pathway 20.

*Ctnnb1* exon 3 encodes part of the beta‐catenin protein domain that when phosphorylated targets beta‐catenin for degradation within the cell. Point mutations, as generally seen in human tumors, and excision of this region in the GEMMs is predicted to extend the half‐life of the beta‐catenin protein leading to nucleo‐cytoplasmic accumulation and transcription of WNT pathway target genes 15, 20. Surprisingly in human ACP samples, nucleo‐cytoplasmic accumulation of beta‐catenin and downstream activation of the pathway, as evidenced by expression of target genes (e.g. *AXIN2*), is mostly limited to only a small proportion of tumor cells, often correlating with epithelial whorls, sometimes referred to as “clusters”^12^, ^22^, ^25^, ^27^, ^39^. In human tumors, these clusters are often seen at the leading edge of tumor invasion, show loss of epithelial differentiation and express stem cell markers (e.g. CD44), 4, 11, 23, 26. In both GEMMs of ACP, the pituitary gland also shows cell clusters with nucleo‐cytoplasmic beta‐catenin, which are similar to the human clusters 2, 15. Next, we will describe in more detail the two mouse models.

## The Embryonic Model of ACP

The anterior pituitary derives from an invagination of the oral ectoderm known as Rathke's pouch. Lineage tracing has shown that *Hesx1* expressing cells within Rathke's pouch give rise to all the hormone producing cells within the anterior pituitary 15. Using a *Hesx1‐Cre* mouse, exon 3 was deleted from the *Ctnnb1* locus by cre‐mediated recombination in cells of the developing pituitary. The pituitaries of these mice were initially enlarged and dysfunctional. A high proportion of mice died at birth due to enlarged pituitaries causing airway obstruction, however those that survived went on to develop large cystic‐solid pituitary tumors leading to death at around 6 months 15.

Analogous to human ACP tumors, these mice showed isolated clusters of nucleo‐cytoplasmic accumulating beta‐catenin cells in the developing pituitary, despite activation of the cre‐recombinase in all cells within Rathke's pouch (Figure 1A) 15. Increased expression of markers of WNT pathway activation, e.g. *Lef1*, *Axin2* and Cyclin D1 were also limited to these clusters. The murine clusters did not express markers of hormone‐producing cell differentiation and a proportion expressed the pituitary stem cell marker SOX2 15. Activation of the WNT pathway in *Pit1* + ve committed progenitors or differentiated hormone‐producing cells did not lead to cluster or tumor formation, highlighting a need for the tumor‐initiating mutation to occur in an undifferentiated cell type 15.

Using a mouse line reporting WNT pathway activation, Andoniadou *et al*. successfully isolated the cluster cells by flow‐activated cell sorting in the embryonic model and performed expression analysis comparing cluster vs. non‐cluster pituitary tissue 1. This identified the high expression of many secreted factors by the cluster cells, including Sonic Hedgehog (SHH) and members of the FGF, TGFβ and BMP families of growth factors as well as many inflammatory mediators such as cytokines and chemokines 1. These findings were subsequently confirmed in human tumors suggesting a strong homology both histologically and molecularly between mice and human clusters, and supporting the usage of this model in therapeutic testing of drugs against these pathways 1.

Further analysis by immuno‐staining and *in situ* hybridisation showed evidence of paracrine signalling between tumor compartments. This was confirmed in human samples; for example, SHH, a soluble ligand was shown to be expressed in the clusters, whilst its downstream target PTCH1 was expressed in clusters and palisading epithelium 1. The activation of the SHH and other pathways was subsequently described by other groups using both targeted and genome‐wide transcriptional approaches, confirming the suitability of this GEMM to study the pathogenesis of the human tumors and complement more conventional analyses of human specimens 13, 16, 19.

In addition to the molecular similarities, this GEMM shows other similarities to ACP. Murine tumors are frequently cystic, often haemorrhagic with histological areas of micro‐cystic change similar to that seen in the stellate reticulum of the human tumors (Figure 1A) 15. Importantly, there are also differences. The tumors do not calcify and ghost cells or wet keratin are not observed. Similarly, the finger‐like invasions that pose a challenge to treating clinicians are not seen in the mouse model. The reasons underlying these differences are not understood. Calcification may require longer terms than a few months, and although highly similar overall, there are specific anatomical differences between the hypothalamo‐pituitary axis in mice and humans, which may explain the lack of brain invasion in murine ACP 33. In conclusion, the embryonic mouse model is a good genetic tool to study the pathogenesis of the human tumors, discover new pathways and test the effects of their genetic or chemical inhibition in vivo. Needless to say, it is not a good model to study brain invasion or the mechanisms involved in wet‐keratin formation or calcification.

## The Inducible Model of ACP

Building on the embryonic GEMM, oncogenic beta‐catenin was specifically expressed in SOX2 positive adult pituitary stem cells using a tamoxifen inducible, mutated form of cre recombinase (*Sox2‐CreERT2* mouse line). The SOX2 cell population was confirmed to have both self‐renewal and differentiation capacity into all lineages of the anterior pituitary, thus demonstrating that stem cells are contained within the Sox2‐expressing cell compartment 2. Similar to the embryonic model, activation of the WNT pathway in this mice from 6 weeks of age led to the development of undifferentiated tumors (synaptophysin and hormone negative) within the normal pituitary tissue, including the presence of nucleo‐cytoplasmic accumulating beta‐catenin cell clusters 2. Surprisingly, lineage tracing using yellow fluorescent protein (YFP) revealed that the tumors themselves were not derived from these cluster cells and did not carry the activating beta‐catenin mutation, as confirmed by laser capture microdissection and PCR 2. This suggests an apparent non‐cell autonomous mechanism of tumorigenesis (Figure 1B), possibly through the paracrine activities of secreted proteins such as SHH, FGFs, BMPs, TGFB, cytokines among others. This phenomenon is increasingly described in several other cancer model systems and extends the traditional understanding of cancer initiation as a cell autonomous process 28, 31. Further details of the mechanisms underlying these processes and their relation to human cancer are a matter of current study.

Together these models have given valuable insights into the cells of origin and cell signalling pathways activated in human ACP. Specific lessons and further questions raised by the models are summarized in Table 1.

**Table 1:** Summary of lessons learnt and future research questions derived from researching GEMMs of ACP.

Lessons:	Future questions:
Mutation of *Ctnnb1* appears sufficient to induce pituitary tumors analogous to ACP. These mutations must be sustained in an undifferentiated precursor/stem cell whether in the embryo or in the adult 2, 15.	What additional molecular steps (e.g. genetic/epigenetic) are between the formation of the clusters in the embryo and tumors in adult mice?
Clusters are not dividing and express a range of soluble factors and immune system genes. Paracrine signalling between tumor compartments occurs (e.g. SHH pathway) 1, 2.	What is the role of these factors? Are they all required or is there redundancy?
Tumors may develop in a non‐cell‐autonomous manner 2.	Are the *Ctnnb1* mutations present in all tumor cells in human ACP? If so, why is nucleo‐cytoplasmic accumulation only seen in some cells? What is the cell‐of‐origin of the tumor tissue in the inducible GEMM?
Common formation of cysts in human and murine ACP 15.	What is the mechanism of cyst formation in human and murine tumors?

## Preclinical Therapeutic Testing of Novel Therapies for ACP

Studies of both human and mouse tumors have highlighted a number of potentially targetable pathways and processes for which a variety of therapeutic agents are available (summarized in Table 2). The described GEMMs of ACP offer an opportunity for testing novel therapies and can be used in a number of different ways.

**Table 2:** Preclinical therapeutic opportunities for ACP.

	Evidence of dysregulation in human ACP	Evidence of dysregulation in murine ACP	Potential therapeutic targeting
Sonic Hedgehog (SHH) pathway	Up‐regulated in gene expression studies of ACP. SHH expressed by clusters with downstream targets also expressed in palisading epithelium 1, 16, 19	Expressed by clusters with targets expressed in non‐cluster pituitary tissue 2.	Preclinical trial using smoothened inhibitor vismodegib in progress 3.
Epidermal Growth Factor Receptor (EGFR)	EGFR is activated (phosphorylated) in ACP clusters 24	Ligand EGF up‐regulated in mouse models. Pathway activation to be confirmed 34	Inhibition by gefitinib reduces ACP cell migration and increases radiosensitivity in primary cell culture 24, 41.
Inflammation	Inflammatory infiltrate observed histologically. High levels of inflammatory mediators (e.g. IL6, α‐defensins) identified in cystic fluid High levels of CXCR4 and CXCR12 correlated with recurrence 17, 32, 37.	Expression of cytokines (e.g. IL1A) by murine clusters. CXCR4 expressed by clusters with ligand CXCL12 expressed by non‐cluster cells 1.	Mechanism of action of intracystic IFNα therapy currently unknown. Specific (e.g. anti‐IL6) or non‐specific (e.g. NSAIDS) immune modulators readily available for testing.
Other	A range of other pathways, e.g. BMP, FGFs, TGFβ, MMPs have been shown to be expressed or activated in murine and human ACP 1, 19.		

The embryonic model is so far the better characterized of the models. Its biology in the early stages of tumorigenesis is being increasingly well‐defined using both targeted assessments of clusters at embryonic and early postnatal stages as well as transcriptome‐ and exome‐wide assessment of both clusters and whole tumors. This increasing knowledge of the model combined with the ability to treat at a relatively young age, without the need of tamoxifen injection makes it an appealing preclinical tool to use for drug testing.

The impact of agents on clusters can be assessed quickly: (i) *in vivo* through administration either *in utero* or early postnatal life; and (ii) *ex vivo* through assessment in culture conditions. To assess the impact on tumor formation, mice can be chronically treated postnatally and followed longitudinally. However, these approaches may represent better assessment of tumor prevention, rather than cure of late stage tumors required by patients. Nonetheless, the identification of drugs capable of preventing tumor growth in the mouse model may be relevant if these pathways are still important in well‐developed human tumors. The use of a human ACP xenograft mouse model could extend the results obtained from the preclinical research using the GEMM.

Late stage tumors in this model develop slowly and unpredictably, over a prolonged period of several weeks or months (Current median survival 23 weeks, inter‐quartile range 13–34 weeks, *n* = 93) (unpublished). In addition, the biology of the well‐developed mouse tumors is less understood. Mice are usually humanely culled due to symptoms of hydrocephalus, often relating to haemorrhage into cysts, which makes design of studies and end‐points challenging. Such studies will likely require preclinical imaging assessments to both establish when to treat and also to assess treatment response. Novel analyses aiming to understand the imaging characteristics and further refine the growth pattern of tumor formation in these ACP mouse models are currently underway 8.

It is increasingly recognized that preclinical trials should as far as possible recapitulate human treatment regimens 14. For ACP the standard treatment is usually surgical resection, and then if resection is incomplete, radiotherapy of 56Gy in multiple fractions of 1.8‐2Gy. The location of the GEMM tumors within the sellar makes surgery unlikely to be feasible in these murine models, however, technology to deliver stereotactic radiotherapy to mice is increasingly available and it is hoped that this can be incorporated for analyses of at least some therapeutics in the GEMMs of ACP 21.

## Conclusion

The two GEMMs of ACP have given many novel insights into ACP biology, which have been subsequently validated in human tissue. In addition, they have highlighted many further areas of study and generated novel hypotheses, for which they are well placed to test. The common molecular aetiology together with the proven capacity to predict and identify novel genes/pathways make these models suitable to study human ACP. As highlighted, they also have limitations and they only model certain aspects of the disease. As the molecular landscape of human ACP becomes better defined it is likely that further refinement of these models will also be required. Whilst *CTNNB1* to date is the only recurrently mutated gene identified in ACP, other additional alterations, whether genetic or epigenetic, may be required for development of the human invasive disease. Once identified these will also require mouse modelling. Similarly, no GEMM of PCP has been developed so far. Such a model would be of help in refining the role of BRAF inhibitors and in solving the controversy of the cell‐of‐origin of the craniopharyngioma subtypes. Recent advances in the field of transgenesis, such as the use of the CRISPR/Cas technology will facilitate tremendously the generation of new GEMMs 40.

To maximize their potential, GEMMs should be used in conjunction with other approaches. For successful development of any novel therapeutics, a combination of *in vitro* and *in vivo* disease models, using both GEMMs and xenografts, will be required to ensure that all the different aspects of the biology and pathogenesis are collectively covered. Similarly, a combination of both genetic and therapeutic targeting approaches will likely give the best level of preclinical efficacy of specific pathways. The advances in the understanding of human ACP for the last 10 years have been phenomenal and we anticipate that specific target therapies should be implemented in the next 10 years ahead.
